# A Straightforward Method for Copper Determination in Fish Samples via Direct Solid Sample Analysis Line-Source GFAAS

**DOI:** 10.1021/acsomega.4c08054

**Published:** 2025-02-28

**Authors:** Matheus Fernandes Filgueiras, Marie Novotná, Michaela Vašinová Galiová, Aderval S. Luna, Jefferson Santos de Gois

**Affiliations:** † Rio de Janeiro State University, Graduate Program in Chemical Engineering, Rua São Francisco Xavier 524, Rio de Janeiro 20550-013, Brazil; ‡ Brno University of Technology, Institute of Chemistry and Technology of Environmental Protection, Faculty of Chemistry, Purkyňova 118, Brno 61200, Czech Republic; § Rio de Janeiro State University, Department of Analytical Chemistry, Rua São Francisco Xavier 524, Rio de Janeiro 20550-013, Brazil

## Abstract

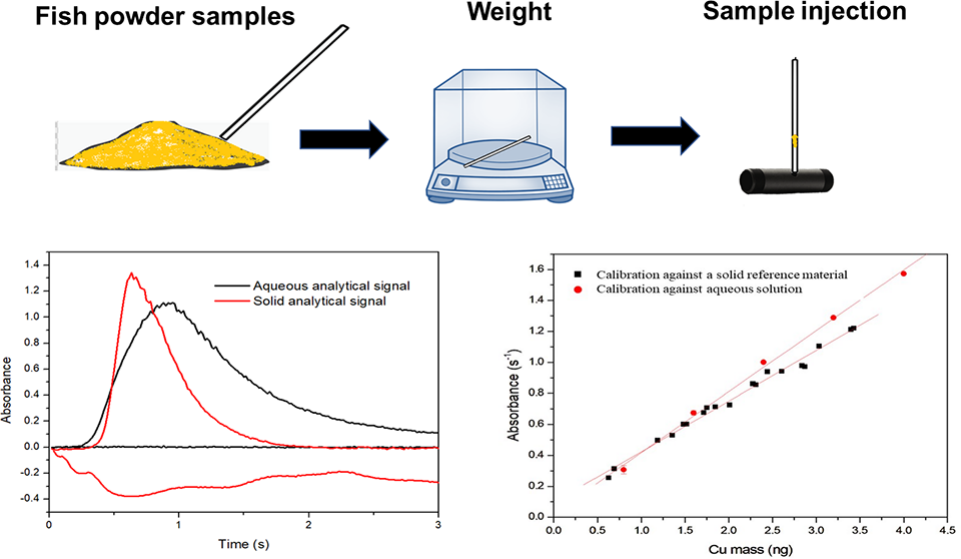

This manuscript describes a simple and reliable method for direct solid sample analysis applied for Cu determination in powdered fish samples using line-source graphite furnace atomic absorption spectrometry. Direct solid sample analysis was performed using a laboratory-made device based on glass capillary tubes for sample mass measurement and transportation to the graphite furnace. The pyrolysis and atomization temperatures were optimized; the optimum conditions were achieved at 1100 °C (pyrolysis) and 2400 °C (atomization). External calibration against aqueous solutions proved to be a reliable alternative for Cu quantification in fish samples with good accuracy. The detection and quantification limits for Cu determination in fish tissues were 0.04 ng g^–1^ and 0.12 ng g^–1^, respectively. The proposed method revealed good sensitivity while being simple and reliable.

## Introduction

Copper pollution in coastal areas is a global environmental problem of public health concern, mainly due to the use of this element in antifouling coatings for ship hulls.^1^ Other sources of Cu pollution are sewage and civil and industrial effluents;^2^ therefore, Cu pollution needs to be monitored.^3^

Toxic elements, such as Cu, can accumulate in fish tissues by being taken up either via the food chain or by penetrating cell membranes to reach the cell nucleus, where they bind to cellular proteins. Therefore, fish species can be used as biological indicators of contaminants, especially because they are at the top of the food chain and can accumulate these elements.^4^ Contamination of fish samples with metals has been reported for sardine,^5^ tuna,^6^ and hake.^7^

In the case of Cu, determination in fish samples can be performed by a variety of analytical techniques that require at least one sample preparation step to solubilize the analyte in a medium suitable for analysis. This can be done, among other methods, by microwave-assisted digestion of the samples with inorganic acids (HNO_3_, HCl, or HF), sample preparation in alkaline media, or by dry ash methods.^8−10^ Special attention is paid to graphite furnace atomic absorption spectrometry (GFAAS) due to its high sensitivity^2,11−13^ and the possibility of slurry sampling^14^ and direct solid sample analysis.^15−17^ These approaches are useful when dealing with relatively complex sample matrices.

The advantages of direct analysis of solid samples are the high sample throughput, high detection capability (as no sample dilution is carried out), relatively low costs for the analysis, and lower risk of analyte losses. In addition, it can be particularly useful for complex sample matrices that are not easy to dissolve.^18,19^

Copper determination via direct solid sample analysis can be accomplished using high-resolution continuum source graphite furnace atomic absorption spectrometry (HR-CS GFAAS). In this technique, the solid sample is introduced into the graphite furnace and a heating program is applied. The first step is the drying step (to remove solvents from the sample), the second step is the pyrolysis step (which is important to remove the sample matrix, thus reducing interferences), and finally, the atomization step (where the analyte is atomized and the analytical signal is collected).^20,21^

In commercially available devices for direct solid sample analysis (available only for HR-CS GFAAS), the samples are weighed on graphite platforms and placed in the graphite furnace for further analysis. Direct analysis of solid samples using HR-CS GFAAS has been successfully used for direct analysis of solid biological samples,^21^ such as geological,^22^ food,^23,24^ fuel,^25^ and polymer samples.^26^

One of the main advantages of HR-CS AAS is the high-resolution spectra, which enable better correction of spectral interferences. Unfortunately, an HR-CS AAS is not available in most laboratories (and it is only offered by one instrument manufacturer in the world); therefore, a line-source GFAAS is the most common instrument in analytical laboratories. Since line-source GFAAS does not provide high-resolution spectra, direct analysis of solid samples using this technique has not yet been thoroughly explored, posing significant challenges from instrumentation for placing samples in the graphite furnace to correcting for spectral interferences.

Therefore, in this work, we present a cost-effective strategy for the direct analysis of solid samples with a line-source GFAAS, which was developed and applied for the determination of Cu in fish samples from the coastal area of Rio de Janeiro, Brazil.

## Experimental Section

### Instrumentation

All measurements were carried out using a line-source atomic absorption spectrometer, model A Analyst 300 (PerkinElmer, USA), equipped with an electrothermal atomizer model HGA-800 and a background correction system (deuterium lamp). The cathode lamp, also from PerkinElmer, was operated under the recommended conditions, and the monitored wavelength was set at 324.8 nm. The measurements were performed by using the integrated peak area.

For the 3D spectrum, a ContrAA 800 (Analytik Jena, Germany) high-resolution continuum source spectrometer was used. It is equipped with a Xe short-arc lamp as a continuum light source combined with a high-resolution double-Echelle monochromator and a charge-coupled device detector. Graphite tubes with a pin platform were utilized for the analysis.

Inductively coupled plasma optical emission spectrometry (ICP-OES) analysis was carried out using an instrument model iCAP 6000 (Thermo Scientific, USA), monitoring the wavelength of 324.754 nm. The ICP-OES was equipped with a V-Groove nebulizer and a cyclonic spray chamber. The operating parameters used were radial view, pump rate (60 rpm), plasma gas flow (12 L min^–1^), radio frequency power (1300 W), auxiliary gas flow rate (1.0 L min^–1^), and nebulizer gas flow rate (0.4 L min^–1^). All measurements were performed in triplicate. Argon with a minimum purity of 99.95% (Air Liquide, Brazil) was used as the main, auxiliary, and nebulizer gas for ICP OES.

Microwave-assisted digestion was performed using a microwave oven model “Microwave Reaction System, Multiwave PRO” (Anton Paar, Graz, Austria), with closed digestion vessels, operating at a maximum pressure increase rate of 0.5 bar s^–1^; maximum microwave power of 1200 W; maximum internal temperature of 200 °C; and maximum pressure of 20 bar.

A drying oven model SSAi–110 l (7 Lab, Brazil) and a 200-mesh sieve from Bertel, Brazil, were used for sample preparation. Capillary glass tubes (Perfecta, Brazil), with an internal diameter of 1 mm, were used to weigh and transfer the solid sample to the graphite furnace. An analytical semimicrobalance model AUW220D (Shimadzu, Japan) with a precision of 0.01 mg was used to weigh mass aliquots into the capillary tubes.

#### Samples and Sample Preparation

Seven fish samples (croaker, tuna, viola, hake, dogfish, pink conger, and sardine) were obtained from a local market in Niteroi, Rio de Janeiro. The raw fish fillets were first dried in a drying oven at 95 °C for 72 h, milled with an electric grinder, and sieved to 200 mesh. Aliquots of 0.1 to 1.1 mg of the pulverized samples were weighed into the glass capillary tubes and manually introduced into the graphite furnace.

Two certified reference materials (CRMs) were used to assess the accuracy of the method: fish protein powder (DORM-5) from the National Research Council Canada and fish muscle powder (ERM-BB422) from the European Reference Material (Belgium). The aqueous Cu standard solution (SpecSol, Brazil) with a concentration of 1000 mg L^–1^ was used for external calibration against aqueous solutions.

Microwave-assisted digestion was performed by measuring 100 mg of each sample directly into the polytetrafluoroethylene flask of the microwave oven, and then, 3.0 mL of HNO_3_ and ultrapure water were added to complete the final volume of 6.0 mL. The vessels were closed and subjected to the following heating program: heating to 200 °C for 8 min, maintaining at 200 °C for 14 min, and cooling to 65 °C for 23 min. The samples were transferred to a 50.0 mL polyethylene flask and diluted for further analysis.

#### Direct Copper Determination by GFAAS

For direct solid analysis, the pulverized solid samples were weighed into capillary glass tubes and transferred to a graphite furnace with the aid of polyamide rods. External calibration against solid standards was carried out with the CRM DORM-5, and external calibration against aqueous standards was performed with solutions of 0.8–4 mg L^–1^ Cu (using aliquots of 20 μL). The optimized graphite furnace heating program is described in Table 1.

**Table 1:** Temperature Program for Cu Determination in Fish Samples by Graphite Furnace Atomic Absorption Spectrometry Using Direct Solid Sample Analysis

Step	Temperature (°C)	Ramp (s)	Hold (s)	Argon Flow (L min^–1^)
Dry	80	1	5	0.250
Dry	120	1	10	0.250
Pyrolysis	1100	1	5	0.250
Atomization	2400	0	5	0
Cleaning	2600	1	5	0.250

#### Data Analysis

Mean values, standard deviation, and relative standard deviation were calculated using Microsoft Excel, and statistical tests were carried out using the software R (v. 4.3.3).

## Results and Discussions

### Pyrolysis and Atomization

**Figure 1:**
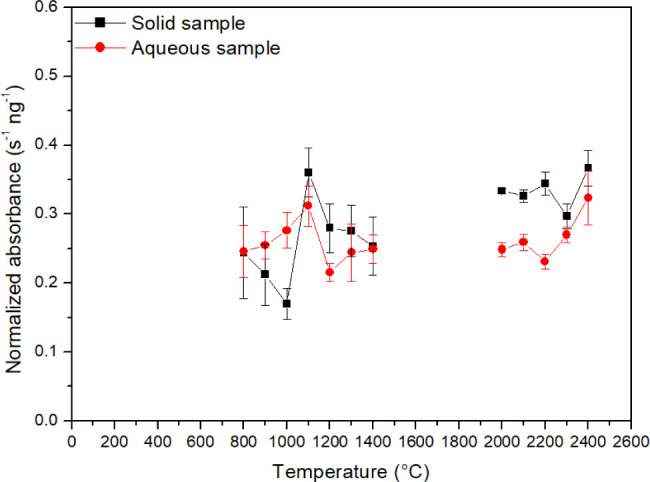
Pyrolysis and atomization curves for aqueous Cu solution (at the same concentration for all temperatures) and standardized absorbance of the solid fish sample determined by GFAAS.

Pyrolysis and atomization temperatures (Figure 1) were determined for aqueous solutions (20 μL of a 0.2 mg L^–1^ Cu solution) and a powdered fish sample (average mass of 0.5 mg), without chemical modifiers. The analytical signals were normalized by dividing the integrated signals by the Cu masses. Pyrolysis temperature was investigated at an atomization temperature of 2200 °C, while the atomization temperatures were studied at a pyrolysis temperature of 1100 °C. The optimum temperatures for both media (solid and aqueous) were reached at 1100 and 2400 °C for pyrolysis and atomization, showing similar thermal behavior in both media (aqueous and solid). This similarity could indicate that the solid fish matrix did not affect the thermal behavior of the analyte, and this element is a good candidate for direct solid determination without the use of modifiers by GFAAS, which simplifies the analytical method.

#### Spectral and Nonspectral Interferences

**Figure 2:**
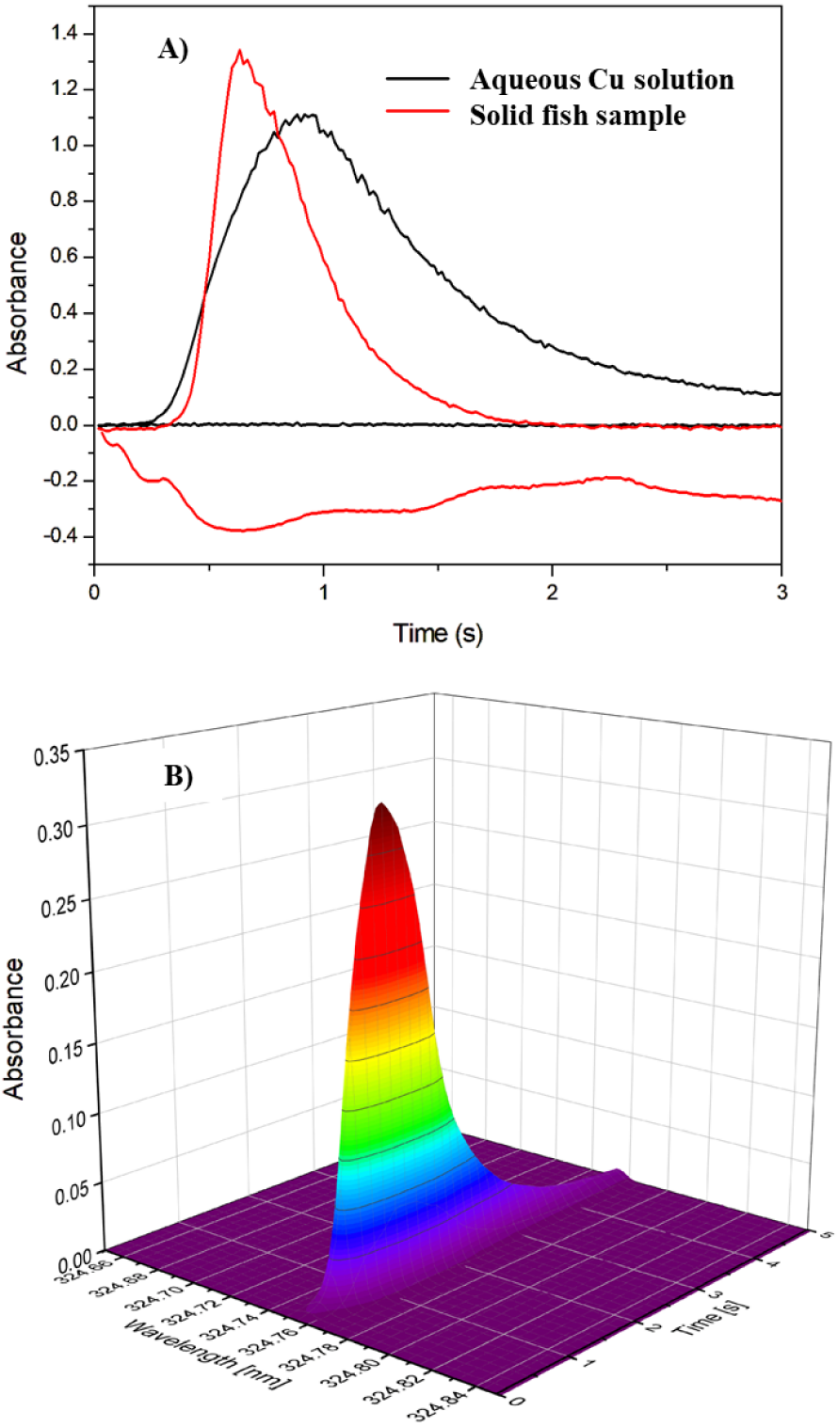
Analytical signals for Cu in fish samples obtained by the direct solid sample analysis via (A) line-source graphite furnace atomic absorption spectrometry and (B) high-resolution continuum source graphite furnace atomic absorption spectrometry.

The analytical signals were recorded with a line-source GFAAS and the HR-CS GFAAS to investigate the presence of a complex background that could jeopardize the measurements (Figure 2). Figure 2A shows the analytical signals obtained from a line-source GFAAS for aqueous solutions and the solid fish sample, and Figure 2B shows the 3D analytical signal obtained for the solid fish sample from an HR-CS GFAAS.

**Figure 3:**
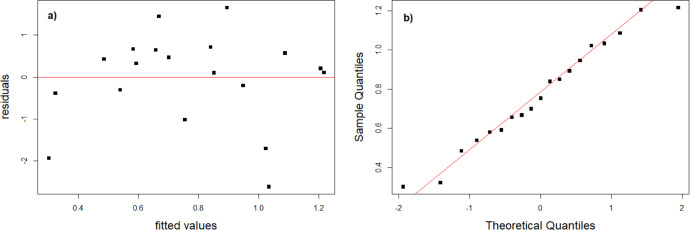
Linearity test for calibration against solid standards for Cu determination using a line-source GFAAS. (A) Statistical residuals graphics for solid calibration and (B) theoretical vs obtained concentrations using a linear regression.

The analytical signals obtained with the line-source GFAAS did not show a complex background. Therefore, the use of a simple deuterium lamp for background correction can be a good alternative when other techniques are not available. In addition, the 3D signals obtained from the samples using an HR-CS GFAAS (Figure 3B) confirm that no complex background is expected in the investigated samples. Although a similar thermal behavior was observed for aqueous and solid standards, the signal profiles differed only slightly. However, since the integrated signal was used, this small profile difference did not affect the accuracy of the method when calibrated against aqueous standards (as will be further shown).

As the direct solid sample used in this work was achieved by a new technique where the powdered samples were weighed into capillary tubes and introduced into the graphite furnace, it is important to understand whether linear regression can be applied to the analytical calibration curve; therefore, linearity tests were performed. First of all, it was investigated whether the residuals of the linear regression follow a normal distribution. Figures 3A and 3B show the residuals for the models with the direct solid sample analysis. It can be seen that a normal distribution is expected in both cases, which was confirmed by the Shapiro–Wilk test. The homoscedasticity was confirmed with the Breusch–Pagan test , and the autocorrelation hypothesis was negated with the Durbin–Watson test. All statistical tests were performed considering *p* = 0.05.

**Figure 4:**
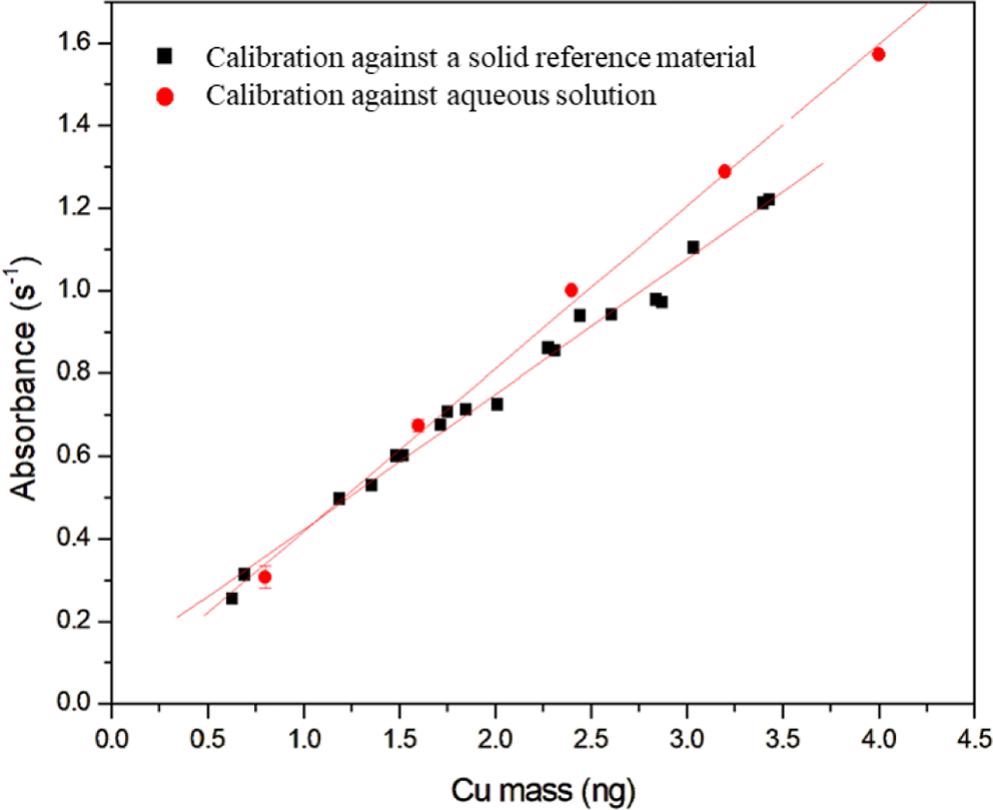
Analytical calibration curves for Cu determination in fish samples using a line-source GFAAS. Red circles represent the calibration against aqueous standards, and the black squares represent the calibration against the solid certified reference material DORM-5.

To investigate nonspectral interferences in Cu determination with a line-source GFAAS, two analytical curves were generated to compare the sensitivity (Figure 4). One analytical calibration curve was against aqueous standards and the other was against a certified solid reference material (DORM-5). For the calibration against a certified solid reference material (Cu masses from 0.3 to 3.3 ng), 0.1–1.0 mg of solid sample aliquots were measured. For calibration against aqueous standards, a Cu solution of 400 μg L^–1^ was prepared, and the volumes of 4, 8, 12, 16, and 20 μL of this solution were analyzed with an automatic sampler (Cu masses from 0.8 to 4 ng).

Both analytical curves (Figure 4) showed a similar slope, which was confirmed by a *t* test (α/2 = 0.05). Therefore, a similar sensitivity was obtained, indicating that the same behavior of Cu is expected when analyzing fish samples or aqueous standards, and therefore, no nonspectral interferences were observed.^19,27^ The same behavior of the analyte was also observed for pyrolysis and atomization temperatures, underlining that Cu does not suffer from nonspectral interferences when analyzing fish samples by direct solid analysis using GFAAS, even without the use of modifiers.

#### Analytical Figure of Merit

The limit of detection (LOD) and the limit of quantification (LOQ) were calculated by using the “zero mass response” technique, which consists of repeating the analysis of the complete cycle of the temperature program without a sample on the nebulizer (empty graphite furnace). The LOD was calculated using the standard deviation of ten measurements of the blank sample, multiplied by 3.3 and divided by the slope of the analytical calibration curve, and the LOQ was calculated by multiplying the standard deviation by 10.^35^

The analytical figures of merit for the determination of Cu by direct solid analysis in a line-source GFAAS are shown in Table 2. The LOD obtained was lower than the values reported in the literature for the determination of Cu in fish samples by ICP-MS (0.05 μg g^–1^),^28^ ICP-OES (0.002 mg g^–1^),^29^ GFAAS after microwave-assisted digestion (0.083 μg g^–1^),^30^ and FAAS (0.00374 mg g^–1^);^31^ it was also lower than in other studies that analyzed different sample matrices.^32,33^

**Table 2:** Analytical Figure of Merit for Cu Determination in Fish Samples by Line-Source GFAAS Using Direct Solid Sample Analysis

Parameter	Values
Calibration curve range (ng Cu)	0.8–4.0
Slope (s^–1^ ng^–1^)	0.3933
Intercept (s^–1^)	0.0227
*r*^2^	0.994
LOD (ng g^–1^)	0.04
LOQ (ng g^–1^)	0.12
Short-term precision % (*n* = 10)	0.5
Blank mean (s^–1^) (*n* = 10)	0.017
Blank range (s^–1^) (*n* = 10)	0.014–0.019

The accuracy of the method was evaluated by analyzing certified reference materials. Table 3 shows the results for the proposed method and the reference values, indicating that the results are in agreement with the certified values, as shown by a *t* test (95% confidence interval) using the analytical calibration against aqueous standards.

**Table 3:** Evaluation of the Accuracy of the Method Using Certified Reference Materials Labeled with the Cu Concentration Compared to the Linear Regression Equation from the Aqueous Calibration with a Confidence Interval of 95%, Using GFAAS

Sample	Certified (mg kg^–1^)	Obtained (mg kg^–1^)
ERM-BB422	1.67 ± 0.16	1.63 ± 0.10
DORM-5	3.30 ± 0.07	3.32 ± 0.40

#### Copper Determination in Fish Samples

Six fish samples, collected in Niteroi, Rio de Janeiro, were analyzed using the proposed method and by ICP-OES after microwave-assisted digestion for comparison purposes (Table 4). The homogeneity factor (*H*_e_) was calculated according to Kurfurst,^35^ where values of *H*_e_ < 10 mean that the sample can be considered homogeneous for the studied analyte. The results from the proposed method are in agreement with the proposed method using a *t* test at a 95% confidence interval, demonstrating the accuracy of the proposed method. Despite most samples presenting as homogeneous within the mass range studied (about 1 mg), tuna samples presented as heterogeneous, and most values obtained for the other samples are close to 10, which may suggest that homogenization of the samples prior to analysis may be critical. Copper concentration in the samples ranged from 0.62 to 2.10 mg kg^–1^ (Table 4); canned sardines may present Cu concentration in the range of 0.62–2.62 mg kg^–1^.^29^ For those obtained directly from fisheries, a mean concentration of 1.8 ± 0.6 mg kg^–1^ was observed.^5^ Moreover, concentrations of this element in tuna samples have been reported as 0.62 mg kg^–1^ for canned samples^6^ and 1.71 mg kg ^–1^ for fresh samples.^34^ Hence, it is possible to conclude no Cu contamination in the studied samples.

**Table 4:** Copper Concentration in Fish Samples Determined by Direct Solid Sample Analysis Using Line-Source Graphite Furnace Atomic Absorption Spectrometry and Inductively Coupled Plasma Optical Emission Spectrometry (95% Confidence Interval, n = 6 for SS GF AAS, n = 3 for ICP-OES)

Sample name	SS GF AAS (mg kg ^–1^)	ICP-OES (mg kg ^–1^)	H_e_ (% mg^1/2^)
Viola	0.91 ± 0.02	0.93 ± 0.02	7.5
Tuna	0.62 ± 0.16	0.66 ± 0.11	18.6
Dogfish	0.97 ± 0.03	0.95 ± 0.17	9.9
Pink Conger	1.03 ± 0.02	1.03 ± 0.10	9.3
Sardine	2.03 ± 0.01	2.10 ± 0.14	3.9
Hake	1.17 ± 0.04	1.25 ± 0.17	9.4

## Conclusion

In this work, we demonstrated the feasibility of the direct analysis of solid samples using a simple GFAAS line source. Although there are no commercially available devices for direct solid sample analysis with a line-source GFAAS, we have shown that it is possible to develop reliable and straightforward methods with this device. It was possible to measure sample masses of less than 1 mg and to perform direct analysis of solid samples in GFAAS (when no commercial instrument is available) using capillary glass tubes. Direct Cu determination in solid fish samples could be performed with a line-source GFAAS without the use of chemical modifiers, with lower detection and quantification limits than in other work in the literature. Calibration against aqueous standards showed the same sensitivity with good precision and accuracy compared with calibration against solid standards.
